# A Time Tree Medium Access Control for Energy Efficiency and Collision Avoidance in Wireless Sensor Networks

**DOI:** 10.3390/s100402752

**Published:** 2010-03-26

**Authors:** Kilhung Lee

**Affiliations:** Department of Computer Science and Engineering, Seoul National University of Technology, 172 Gongnung2-dong, Nowon-gu, Seoul, 139-743, Korea; E-Mail: khlee@snut.ac.kr; Tel.: +82-2-970-6704; Fax: +82-2-977-9441

**Keywords:** sensor network, time tree scheduling, media access control

## Abstract

This paper presents a medium access control and scheduling scheme for wireless sensor networks. It uses time trees for sending data from the sensor node to the base station. For an energy efficient operation of the sensor networks in a distributed manner, time trees are built in order to reduce the collision probability and to minimize the total energy required to send data to the base station. A time tree is a data gathering tree where the base station is the root and each sensor node is either a relaying or a leaf node of the tree. Each tree operates in a different time schedule with possibly different activation rates. Through the simulation, the proposed scheme that uses time trees shows better characteristics toward burst traffic than the previous energy and data arrival rate scheme.

## 1. Introduction

Recent developments in microelectronics have produced low-power, low-cost, high-performance sensor nodes with sophisticated communication facilities. These devices can gather information about their surrounding environments once they have been deployed in small or large areas. These are generally referred to as Wireless Sensor Networks (WSN) [1].

One of the most important constraints in sensor networks is energy capacity. Usually, a sensor node is deployed in a broad area with a small battery attached to it. Sometimes, the node can receive its power from the environment (such as solar power), but more often, nodes are energy-bound. In such conditions, a more energy efficient sensor network is required to effectively overcome the energy problem.

Also, as many nodes are deployed in a large area and many geographical or organic obstacles can be encountered in sensor network environments, flexibility and reliability become increasingly important [2]. For instance, the weather can change frequently, and a new obstacle can affect the operating status of the node, or any other adverse condition, resulting in the connections between nodes to be on and off sometimes. Additionally, for dense networks where many sensor nodes are deployed to monitor a target more precisely, a faster and highly reliable data delivery mechanism is required. However, it is not easy to gain both energy efficient and reliable sensor networks simultaneously.

For saving the energy of the sensor network, we can use an energy efficient routing [3], an energy efficient medium access control [4], or both [5]. Also, many versions of managing radio activity to conserve energy are introduced. In this paper, a medium access control and a wakeup scheduling scheme for sensor networks are introduced. This scheme is energy efficient because it effectively reduces the operational energy by controlling the activation schedule of the sensor node. In addition, the scheme provides a traffic distribution and backup route mechanism for efficient energy use in sensor nodes. This scheme is based on some of the previous sleep-based slot protocols for WSNs.

## 2. Related Works

### 2.1. Wireless Medium Access Protocol

A wireless sensor network shares the radio channel and thus, requires medium access control (MAC) for the safe delivery of data between nodes. The MAC protocol decides when competing nodes may access the shared medium and attempts to ensure that no two nodes interfere with each other’s activity. For wireless sensor networks, two approaches are commonly used in wireless access: a contention-based and a schedule-based MAC protocol.

A contention-based MAC protocol can access a shared medium randomly and have a mechanism to avoid collision. ALOHA [6] and CSMA/CA (Carrier Sensing Multiple Access/Collision Avoidance) protocols are examples of such cases. The IEEE 802.11 wireless standard [7] uses the RTS/CTS (Request to Send/Clear to Send) mechanism to avoid collisions and to eliminate the hidden node problem. It includes two schemes known as a distributed coordination function (DCF) and a point coordination function (PCF). The DCF uses a CSMA/CA protocol with acknowledge (ACK). In PCF, a central access point coordinates medium access by periodically polling the other nodes for data. The IEEE 802.15.4 standard designing for use in low rate wireless personal area networks (LR-WPAN) is also a contention-based protocol [8].

Another approach in wireless access is a schedule-based MAC protocol. In this scheme, a wireless node accesses the shared medium in a deterministic fashion. Under a proper scheduling policy, this scheme shows good performance with high traffic loads. TDMA (Time Division Multiple Access) or FDMA (Frequency Division Multiple Access) and CDMA (Code Division Multiple Access) techniques are a good example. However, in most cases, these techniques are used with contention-based techniques for efficient use of the shared medium.

### 2.2. Energy Efficient Medium Access Control Protocol for the Sensor Networks

To reduce the energy of a sensor network, it is essential to manage the radio activity to conserve the energy of a node. This scheme puts the radios into sleep mode either periodically or whenever possible when a node is neither receiving nor transmitting. This scheme can be categorized into two classes: asynchronous and synchronous. In an asynchronous scheme, a node wakes up and checks another nodes’ activity to send or receive data. In a synchronized scheme, a node is activated in a synchronized manner with a neighbor node and starts to send data immediately after activation.

Asynchronous wakeup solutions do not suffer from synchronization overhead, and each node consumes lower power than a synchronous solution. El-Hoiydi [9] introduced a mechanism for waking up sleeping radios asynchronously in ALOHA and named it Preamble Sampling. Hill and Culler [10] used it in CSMA and named it Low Power Listening (LPL). In these schemes, the sender transmits a long preamble and the receiver periodically wakes up and senses the channel. If no activity is found, it goes back to sleep again. If activity is sensed, the receiver will change to its fully active receiving mode.

LPL with preamble sampling has one serious shortcoming. The long preamble can cause throughput reduction and energy waste for both sender and receiver. The WiseMAC protocol [11] builds upon preamble sampling to overcome it. By using additional contents of an ACK frame, each node can learn the periodic sampling times of its neighbor nodes. It uses this information to send a shorter wakeup preamble at just the right time. The frames in WiseMAC also contain a bit which the transmitter uses to signal to the receiver if it needs to awake a little longer in order to receive additional frames intended for it. In Receiver-Initiated MAC (RI-MAC) [12], the receiver sends out invitation beacons at regular intervals. The sender must wait until it sees one and respond by sending the message. Collisions are detected at the receiver, who then sends out a new beacon specifying a contention window, increasing its length on consecutive collisions.

The Reconfigurable MAC Protocol (Berkley MAC, B-MAC) [13] has a limited set of core functionalities and an interface that allows the core components to be turned on/off and configured depending on application needs. The core of B-MAC consists of lower-power listening (LPL), clear channel assessment (CCA), and acknowledgement (ACK). The B-MAC can use the channel sampling of the transmitter node, backoff mechanism, and data exchange acknowledgement. The simplicity, efficiency, and configurability of B-MAC can be used in many practical fields and other complex protocols can be implemented over this B-MAC protocol. X-MAC [14] addresses the overhearing problem associated with LPL’s long preambles by using a strobed sequence of short packets, including the target ID, allowing for fast shutdown of other nodes and receiver response. X-MAC’s shortened preamble approach significantly reduces energy usage at both the transmitter and receiver, reduces per-hop latency, and offers additional advantages, such as flexible adaptation to both bursty and periodic sensor data sources.

The other category is a scheme that a sensor node wakes up periodically and synchronously with neighbor nodes. A node sends a data without wasting a long time for sampling the activity of the other nodes. In S-MAC [15], all nodes of the network wake up at the same time, send data if necessary, and sleep for a predefined interval. This sleep-and-wake duty cycle provides the network with an energy reduction of the node. The problem of S-MAC is its long data delivery time due to the use of the sleep mechanism. An Adaptive Listening [16] as an extension to the S-MAC scheme adjusts the active periods to the traffic to reduce the sleep latency. Timeout MAC (T-MAC) protocol also introduces an adaptive active period mechanism [17]. The sensor node only listens for a short duration at the beginning of a slot and goes back to sleep when no communication happens. These protocols still show low throughput and long delays.

Data-gathering MAC (DMAC) eliminates data-forwarding delay problems by giving the active/sleep schedule of a node and an offset that depends upon its depth on the tree [18]. This scheme allows continuous packet forwarding because all nodes on the multi-hop path can be notified of the data delivery in progress. Furthermore, DMAC suggests a data prediction mechanism and the use of more to send (MTS) packets in order to alleviate problems pertaining to channel contention and collisions. The use of sequential active/sleep and the MTS mechanism make it possible for DMAC to achieve a significant energy savings and latency reduction while ensuring high data reliability. However, DMAC shows many collisions in a densely deployed network.

In a network where many nodes are deployed per unit area, there is no need for every node to be on simultaneously. Too many active nodes in the same area increase the probability of data collision; and data delivery throughput decreases accordingly. A topology control mechanism restricts the set of nodes that are considered neighbors of a given node. This mechanism controls transmission power in a flat network [19], a coordinating task, by introducing hierarchical networks based on clustering [20,21] or turning off specific nodes for a given time [22].

Many of the sensor protocols are combinations of the above-mentioned mechanisms. It is required some mechanism that has an efficient medium access control, and a topology control and scheduling mechanism for data delivery and an energy efficient operation for the sensor networks at the same time. Self-organization, high performance, robust and failure backup mechanisms are other important characteristics of the future sensor networks.

## 3. Time Tree Medium Access Control

The data paths in the network consist of multiple time trees that have different activation time schedules. Each node of the tree activates according to its depth on the tree and sends data to the BS (base station) node, the root of the tree. A sensor node that wants to send data to the BS node, activates at the predefined activation time and sends data to the parent node of the tree. The data sent in the direction of the parent node travels along the node of the tree activated by the time tree schedule and finally arrives at the BS node. Each branch of the tree activates at a different rate according to the schedule of the time tree that is configured to application traffic requirements.

The exchange of data frame is sequenced by the RTS-CTS-DATA-ACK procedure. Each node uses a back-off algorithm to reduce collision when accessing the wireless channel, reactivates at predefined times later, and retries data transmit when the node fails to send data successfully. This continues until the node successfully ends the data-sending sequence within a predefined retry number.

### 3.1. Time Tree Configuration Algorithm

A time tree is a tree or a group of trees rooted at a target base node. A tree is composed of one or more branches. Basically, the tree configuration is performed by all nodes that operate autonomously and distributedly. For the configuration of the tree, the root node broadcasts tree messages periodically. Each node performs the following operation for the configuration and extension of the tree after receiving the tree message.

**Table:** 

**Node’s Basic Operation for the Configuration of the Tree**
Add the sender node of the tree message to the parents group. Select the best parent node from the parents group. Make a parent-child relationship with the selected parent. Broadcast a tree message to neighbor nodes.

The operation of the node for the configuration of the tree is based on the breadth-first search algorithm. At first, the nodes that can be reached from the root node are searched. These nodes become the first level nodes of the tree. These nodes make the root their parent, and extend the tree by sending a tree message and searching for their children. From this time, nodes will receive many tree messages from their neighbors already configured as a member of the tree. All nodes receiving the tree message select a message, a parent node that has the best condition for sending the data to the root node. After joining to the parent as a child member of the tree, the node broadcasts a tree message. This process continues periodically and finally all nodes in the network are included as members of the tree.

A child node selects its parent node. A child node sends a join message to the chosen parent to make a parent-child relationship for the tree. The most preferred metric when choosing a parent node is the tree level of the parent. The node that has the lowest level is selected as a parent. A tree level is a hop-count from the root node. This criteria eliminates the possibility that a loop may exist along the path to the root node.

The next important metric for selecting the parent is the traffic load. By choosing the light-loaded parent, the node can effectively send the data with the best opportunity to avoid collision and additional delay. This also distributes network traffic among nodes, so the network can reduce the dissipated energy and enlarge the lifetime of the network.

When we know the exact traffic load that the parent had, we can use the load as a metric for the tree configuration. If this metric is not allowed or cannot be used for some other reason, we can use other parameters that reflect this traffic metric. Some of these parameters include the number of children, the number of all descendants, and the number of parents of the same branch. Other parameters, such as the energy required to send data to the root node, the signal strength, or the distance to the parent node, can be useful parameters for the configuration of the tree.

When a parent node is down, the relationship between the parent and the child node is broken. Then, the child node searches another parent node from the parents group as the second procedure described in the node’s basic operation. It is same with the occurrence of an obstacle between nodes, or movement of a sensor node to another place.

### 3.2. Topology of the Tree and Data Sending Cycle

**Figure 1.**
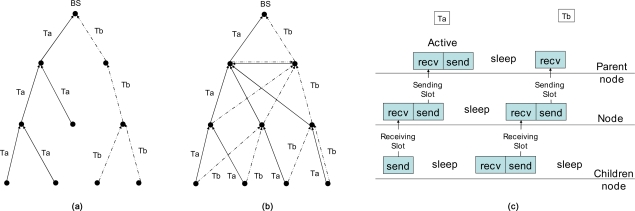
The topology of the time tree and data sending sequences along the tree. (a) Single tree at each node; separated case (b) Multiple trees at each node; folded case. (c) Staggered active cycling along the tree.

**Figure 2.**
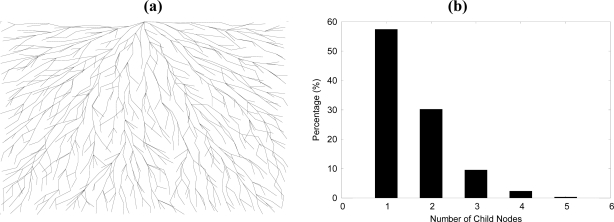
Tree topology of a sensor network. (a) a tree with considering branch identifier. (b) percentage of the number of child node at each parent node.

There are some optional parameters for representing the tree characteristics in a tree message. These include the tree identifier, the branch identifier, tree level, the number of parents in forming a time tree. The tree identifier is a unique identifier of the data sink. A time tree is created per sink node in WSN. The sink node will be a root of the tree that has an identifier and instance of the sink. The identifier of the branch is a number that is given to the node of the first level in a tree. This value is fixed when the first-level node established a relationship with the root node. The number of parents in forming a time tree defines the number of trees that rooted at the originator of the tree message. If this number is one, every node makes a parent-child relationship with only one parent to each tree identifier. Figure 1a is such a case. If this number is two, every node makes two different parent-child relationships to each branch identifier of the same tree identifier. Figure 2b is the result of this scheme. If this number is zero, every node of the network will make a parent-child relationship with each parent that sent a tree message with each different branch identifier of the same tree identifier.

In Figure 1, there is a time tree that consists of two branches or trees, *Ta* and *Tb*, in the network with different branch identifiers of the same sink node (root). In Figure 1a, each node makes one parent-child relationship for each tree identifier. In this scheme, a branch identifier is ignored. Each branch of this tree is separated from each other. Each branch is activated independently, so we can reduce the number of nodes activated at the same time. If we reduce the number of activated nodes, we can reduce the probability of collision and minimize the required energy to send the data to the root. If we can know the exact point of the node location, we can increase the distance between nodes that are activated at the same time. This helps the network lifetime and gains other performances more.

In Figure 1b, the trees *Ta* and *Tb* are folded. The first child of the root node acts as a virtual root of the tree. Each node has a different parent as with different branch identifiers. Each node regards each tree of different branch identifiers as different trees. The advantage of this topology is a distribution of network traffic and provision of an alternate route to the same sink.

The data transmission of a node occurs from the sensor node to the root node sequentially along the tree like DMAC [12]. A child node sends data to its parent node, and the parent node receives the data at the same slot time. Next, the parent node sends the data to its parent node at the next slot of time. This cycle continues to the root node along the tree as in Figure 1c.

After sending data to its parent, the node sleeps until the next scheduled receiving time. The sleep time of a node is more than three times that of an active time [23]. The active time is composed of a receiving and a sending slot time. The distance between nodes differs with a node’s position in the network. So, the safe sleep time to eliminate the interference coming from other node's activity is more than five times the active time [23]. The ratio of an active time with a sleep time is an activation rate. Generally, an activation rate can be less than several percent.

The sending time of a node is determined by a scheduled activation scheme. Each node is activated by the level of the tree and the position of the branch it included. The child node of the root should send data at the receiving slot of the root, and the node should receive the data from its child at one slot before its sending slot.

In general, there is one receiving and one sending slot in one active time; however, the number of receiving or sending time slots can be more than one if required. If one slot is allowed in one active time, one sending slot is followed after one receiving slot. If two slots are allowed in an active time, two consecutive sending slots are followed after two receiving slots. The number of active slots in one frame can be configured differently in each branch or tree. In most cases, it would be a good choice for using one active slot; however, if heavy traffic exists in sensor networks, more consecutive active slot mechanisms would be more efficient, but more than one active slot system adds additional delay per node and thus increases end-to-end latency.

In the other case, you can set the active slot number differently as with the depth of the sensor node in the tree. The traffic gathers and goes to the near root side. It would be more effective if more slots are assigned at the root side of the tree than the far side of the tree. This scheme can lessen the energy of the edge node. Therefore, the edge node can use more energy in sensing and processing the event of its environment than sending or relaying the data.

One simple implementation method of this variable numbers of active slot is by varying the length of a slot. As the length of a slot increases, more than one frame can be sent to a parent node from several child nodes.

### 3.3. Time Tree Scheduling (TTS)

At first, the active slot for data receiving and sending is activated at a minimum rate, called a basic rate. One active slot is activated in a frame, a predefined unit time. As traffic increases, the activation rate is increased twofold. Accordingly, the time tree *Ta* is activated two times in a frame. If the traffic increases more, the activation rate is increased as twofold compared to the current rate. The frame length is not fixed value. It can be varied as with the application environment, from a few seconds to several hundreds of seconds.

The wakeup time *t_w_* (*n,k*) and the starting time of sending slot *t_s_* (*n,k*) of a node *n* in the *i*-th branch with the tree depth of *d* is represented as follows:

(1) $$t_{w}(n,k)=\sum_{k}\{(\frac{k}{r})\tau_{f}+\theta_{i}-d\tau_{s}\}$$

(2) $$t_{s}(n,k)=t_{w}(n,k)+\tau_{s}$$

Here, *k* has an integer sequence value of (–∞, +∞); *τ_f_* is a frame length; *τ_s_* is a slot length; *θ_i_* is a time shift of the *i*-th branch from the starting time of the frame; *r* is a data rate index of the *i*-th branch, and its integer value is 1 when it is the basic rate and *R* when it is the maximum rate. *θ_i_* can be represented as *aτ_s_*, where *a* is an integer value from *0* to the total slot number of a frame, *S*.

Then, (1) and (2) can be rewritten like this:

(3) $$t_{w}(n,k)=\sum_{k}(\frac{k}{r})\tau_{f}+(a-d)\tau_{s}$$

(4) $$t_{s}(n,k)=\sum_{k}(\frac{k}{r})\tau_{f}+(a-d+1)\tau_{s}$$

If *t_s_* (*m,k*) and *t_s_* (*n,k*) are the same for some *a*, *d*, *k* and the node *n* is within reach from the node *m*, then the nodes *m* and *n* are in a same collision area. At this time, RTS and CTS work effectively to avoid collision.

### 3.4. Medium Access Control Operation

The proposed medium access mechanism conforms to the operation of the carrier senses collision-detection and collision-avoidance (CSMA/CA) protocol. The node that wants to send data sends an RTS frame after checking the wireless media and waits for receiving a CTS frame. After successfully receiving the CTS, the node sends a DATA frame and receives an ACK frame of the receiving node. The nodes that want to send data are children nodes, and the receiving nodes are parent nodes. There can be many nodes connected to a parent, so many nodes could be activated at the same time and attempting to simultaneously send data. Also, more than one parent node can be activated at the same time.

When child nodes attempt to send data to their parent, there can be a data collision at the wireless media. This collision causes data-sending failure at children nodes. When there is more than one parent activated together, many children nodes try to send data simultaneously. At this time, the RTS and CTS mechanism resolves the arbitration to seize wireless media. As a result, only one node can successfully send data and others defer to send their data at next activation time.

So, when nodes are densely deployed, many parent nodes will be activated at the same time. To avoid the arbitration of the wireless channel, each branch must activate at different times between nodes. For this reason, when a node selects its parent, choosing a parent that has different branch identifier is ideal. Then, the activation time will be different by the equation (3). In equation (3), a different offset value means a different branch identifier and thus a different activation time. For this, each node should know the branch identifier of its neighbors. This information can be known by the parameters included in a tree message. A tree message has a branch identifier and a depth level of the nodes.

Figure 2 shows the result tree of the sensor network with 1,000 nodes. Figure 2a is case of previous algorithm that choosing a parent with minimum node of same branch identifier. Figure 2b is a percentage graph of number of child nodes at each node. The average number of child nodes of each node is 1.58 and more than 99.5% nodes have maximum 5 children.

A child node receiving a tree message completes the following operation after seeing the parameters of the message: if the node has no parent, the node sends a join request message to the sender of the tree message. If a parent node exists and the computed best parent is different with the current parent, the node sends a join request message to the newly searched best parent. After receiving a join accept message from the best parent, the node broadcasts a tree message. If the parent exists and the computed best parent is same, the node broadcasts a tree message to neighbor nodes.

A parent node receiving a join request message does the following operation: first, the node checks whether the parameter information in the request message is the same as the information it has. If it is the same, the parent node adds the node into a group of children node. Then, the node increases the child number of it and sends a join accept message to the child node. If it is not the same, the node sends a join deny message to the requested node with node parameters of it. The parameters in a join request, accept- and deny-message are same with the parameters in a tree message. It includes a tree and a branch identifier, tree level, parent number, children number and activation rate. After receiving a join deny message, each node does the following operations. First, the node updates the parent information using the information in the join deny message. Then, the node searches a new best parent and sends join request message.

### 3.5. Backoff Algorithms and Retransmission Scheme

Because the wireless channel is shared among all nodes, the node must seize the channel before sending data. The operation of this protocol is based on CSMA/CA. In CSMA/CA, every node checks the media before sending a data. Because there can be more than one node that tries to send data, each node must wait some random time to avoid data collision after the media is free. This waiting time is the backoff time, and it must be different from node to node. In IEEE 802.11 standard [7], the lowest value of wait time is 32 and the highest value is 1024, but in this proposed scheme, there are not many nodes attempting to send data, *i.e.*, around ten or less. Therefore, in our scheme, the maximum value is set to 32 or 127.

At first, each node generates a random number between 0 and 32. Then, each waits the random number of slots before sending an RTS. If the backoff timer expires, the node senses the channel and if the medium is free, the node sends data to the wireless channel. If the medium is busy before the expiration of the backoff timer, the node pauses the backoff timer and resumes it later. At the next activation time, the initial value of the backoff timer is refreshed and restarted again. The node that wants to send data must send an RTS frame and waits for receiving a CTS. If the receiving node does not respond to this RTS, the CTS timer expires at the sending nodes. Then, the sending node retries again by sending an RTS after the expiration of the CTS Timeout.

A data exchange procedure is completed when the node sends a DATA and receives an ACK from the recipient node after seizing the channel by exchanging an RTS and a CTS frame. The failure of a data exchange occurs mainly for three reasons. One reason is a failure to seize the wireless channel. Before sending an RTS, other node can seize the channel by sending an RTS frame. The other reason is the collision of frames. The collision of RTS can lead to non-response of a CTS frame. Then the node sends an RTS again after the CTS timeout. If the CTS retry number goes over the maximum retry number, the node must retry the data sending procedure at next active time. Another reason for data sending failure is if the receiving node is unready to exchange data. The receiving node can be out of power or not wake up in time for some reason. At any rate, the sending node will try to send an RTS until the retry number has reached the maximum retry limit.

The sleep time of a node is long compared with the active time, which means that if a node retries at next scheduled wakeup time, the delay will be very long. So, without waiting for the next normal scheduled active time, the node can retry at some predefined times after the current active time. If a failed node retries directly after the current active time, it will interfere with the data exchange of its parent node. Therefore, the node should retry the data sending procedure after some cycles wait. This retry interval should, at a minimum, be greater than three active times. The suitable value for this could be five or more to avoid data interference.

Normally, each station will sleep until the next normal scheduled time. So, if a node wants to send data during a retry active time, it sends a DATA frame with one more data bit set to one in the Frame Control (FC) field. Then, the parent will know it and respond with an ACK frame with an additional data bit set. If there is no data to send, it sends an RTS with a duration value of zero in the duration field and one more data bit set in the FC field. We call it as a zero RTS or a zRTS frame. The value of the duration field at this zRTS frame reflects only the time value for the receiving of CTS frame. If there is more than one node that wants to get a retrial data exchange, a zRTS arbitration will occur. If a parent receives a zRTS well, it responds with a CTS and will wake up at the retry activation time. After this zRTS-CTS exchange procedure, the current active slot will end without the real data exchange. It is only a sign of demanding retry slot between the child and parent node. The value of zero duration of FC field at an RTS frame makes it possible for other zRTS-CTS procedures right after this exchange procedure.

**Figure 3.**
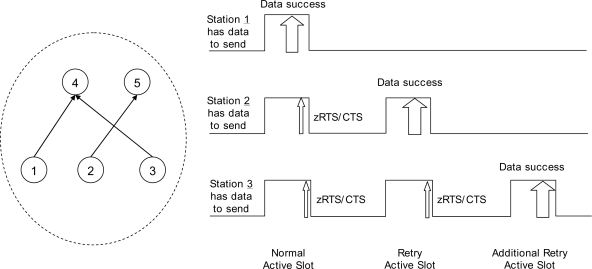
Reservation and retrying procedure between parents and child nodes using zero RTS frame.

Figure 3 shows the operation of data sending at three nodes. They are all on the same level of the tree. Their parents may be the same or different. At the normal wakeup schedule time, station 1 seizes the channel and finishes the data exchange procedure successfully. Stations 2 and 3 send a zRTS frames after the data exchange. There will be an RTS arbitration, and nodes 2 and 3 successfully finish a zRTS-CTS exchange. Nodes 4 and 5 will wake up at the retry active time. At the first retry active slot, station 2 successfully finishes data exchange and station 3 sends a zRTS. Next, at the more retry active slot, station 3 successfully finishes data exchange with its parent.

**Figure 4.**
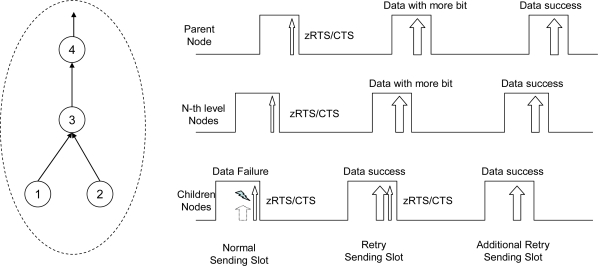
Retransmission procedure between parent and child nodes along the tree using more bit in DATA frame or zero RTS frame.

Figure 4 shows the operation of the data sending procedure along the tree to the root direction. Stations 1 and 2 have data to send, and at the normal sending slot, they fail the data exchange. When there is not enough time in the current sending slot, each node must stop the data sending procedure and start to send a zRTS for the reservation of retry active slot. Stations 1 and 2 exchange zRTS-CTS frames before ending the current slot. Station 3 sends a zRTS frame to its parent for sending data from the children node at the retry active slot. Station 4 relays this zRTS frame to its parent and this continues to the root along the tree. Station 1 finishes data sending at the retry sending slot after successfully seizing the wireless channel. Right afer this data exchange, node 2 sends a zRTS. After node 3 responds to node 2 with a CTS at receiving slot, it sends a DATA to its parent with a more data bit set for the reservation of additional retry slot. In the additional retry sending slot, node 2 sends data to node 3 with a cleared more data bit and the additional active slot ends.

**Figure 5.**
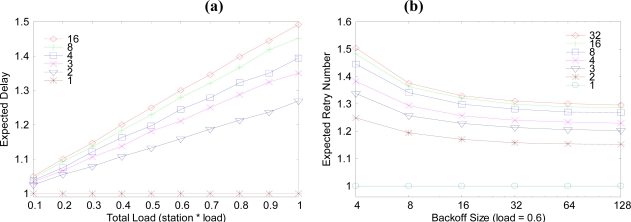
Expected delay and retry number as with load and backoff size. (a) expected delay from the child node to the parent node. (b) expected retry number of data sending from when the traffic load is 0.6.

Figure 5a is a graph of the expected delay from the child node to parent node. In this graph, the child node number of one parent is varied from 1 to 16, and 32. When the child node is one, the expected delay is 1 slot time. As the child node number increases, so do the expected delay and the traffic load. The delay difference between the node numbers 1 and 2 case is bigger than the delay between 2 and 3. So, the one-to-one parent-child case is the best case. Figure 5b explains the expected retry number of data sending procedures between a child and a parent. The difference between 32 and 127 is not much more than expected. It would be more efficient using 3 times retry with maximum 32 backoff slots than using one time retry with maximum 127 backoff slots.

### 3.6. Rate Adaptation to the Traffic Load

The bandwidth of a sensor network can be expressed by its activation rate. At first, every branch of the tree activates at a basic rate. If the traffic load increases, the overflow traffic can be transferred at retry active time. If the number of retries increases, it is more efficient data to send at an increased activation rate than the basic rate with every additional retry. Then, the activation rate is increased twofold. If the traffic increases more, the activation rate is increased twofold again.

By this procedure, the rate can be increased to the maximum rate. If the node has more than one tree to the same target node, the node can distribute traffic to each tree of the time tree. Then, the possible maximum data rate is the sum of data rate of each tree of the time tree.

If periodic reporting is required, the root node can reserve data bandwidth of some branch of the tree. The reservation of the bandwidth begins with root node (centralized) or sensor node (distributed). The root node defines the activation rate of each branch of the tree. If the overall bandwidth is increased, the node increases the activation rate of the branch. A sensor node can increase the bandwidth of the branch along the path from it to the root node. If a request of bandwidth increasing sent from a sensor node to the root direction, each parent increases the bandwidth of the branch by reserving retry active slot as required. If the current bandwidth is 16 kbps and a sensor node reserves 16 kbps more, then one additional retry active slot is scheduled automatically after a normal activation schedule. Certainly, the branch can be activated if more data should be sent after the reserved retry active slot. As a result of the reservation, the activation rate of the branch is different at each path of the branch. In near side of the root, the bandwidth of a branch can be larger than that of the branch of a leaf side.

**Figure 6.**
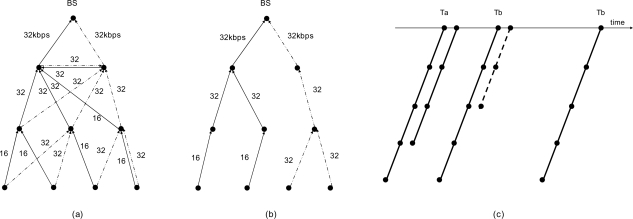
Bandwidth of the branch and activating sequence. (a) folded case. (b) separated case. (c) normal and temporary activating sequence of the branch.

Figure 6 shows the activation rate and the activation cycle. In Figure 6a and 6b, each number represents the activation rate of the path at each branch. This rate is different at each branch and tree. Even in a same branch, the rate is different at the leaf side and the root side. Figure 6c shows a stream of activation events from the leaf to the root side. The solid line represents a reserved cycle and the dotted line represents a retry activated cycle at each node. In Figure 6c, *Ta* and *Tb* have same activation rate. *Ta* has a basic rate and additional activation rate requested by a node in the branch. *Tb* has a doubled activation rate than *Ta* and a temporal retry activated cycle.

### 3.7. Time Synchronization and Routing Function

The RTS and CTS mechanism provide a time to prepare a node to be ready for data exchange. Generally, all nodes of a network must be timely synchronized for successful and correct data exchange. If a node wakes up early or late, the data exchange process can operate abnormally. At this time, the RTS and CTS mechanism provides timing adaptation to this situation.

A routing operation takes place at a source node and a node that detects a node failure of its neighbors. All routing data are destined for each root of the tree. At a source node, a time tree is selected as with the destination of the data. Then, the source node selects a suitable branch of the found tree and the node sends the data to its parent of the branch. Afterward, each data is relayed along the branch of the tree.

While passing along the branch, the next node of the data is found at the MAC layer of each node. All intermediate nodes of the branch operate like a bridge. Each node of the branch is activated at a specific time to send the data to its parent. A node has multiple parents, one for each destination. The activation time is different for each branch of the tree. Even if the activation time is the same, the node can know the parent node by the destination information of the data frame.

If there is a node or link problem in a branch, the node won’t send data to its parent. Instead, the node will send a message to the upper layer, and then the node should refer to the routing table to find a suitable other branch of the time tree targeting to the destination of the data. This operation can occur when there is a heavy congestion when it is impossible to send the data within a permissible delay tolerance. If data is distributed to two trees of the same destination, minimum data can be delivered to the destination even though one of its trees has a problem, and the other branch or tree of the time tree can be used as a backup route to the destination.

## 4. Simulation and Results

For the simulation of the proposed scheme, the ns-2 simulation package is used. To see the characteristics of the proposed scheduling scheme and the operation of the protocol, S-MAC [15], DMAC/MTS [18] and Full Active cases are simulated together. The metrics in this simulation are energy consumed, data transfer delay and the data delivery rate to the normal traffic and burst traffic.

### 4.1. Simulation Environments

The number of sensor nodes in this simulation is 100, except for the BS node. The number of the child of the root node is 10. Each child node forms a branch like in Figure 1a. The number of the branches in network is set to 10. In this simulation, all data from the sensor node are sent to the BS node along the tree. Other simulation parameters are listed in Table 1. When increasing the data generation rate, the collision rate is increased and the error rate also increased. The end-to-end data transfer delay also increased and the overall data transfer performance is degraded. If the number of retransmissions is over a specific number, data is discarded at that node.

For all cases, the activation duty rate is set to ten percent. For S-MAC, DMAC/MTS and the proposed cases, the active time including sending and receiving slot is 19.74 ms. In the simulation of the proposed scheme, the activation rate is fixed with the basic rate and we didn't use the retry and rate control mechanisms. If failure occurs in sending data to the parent node, the node will retry at the next active time.

Five cases are simulated and compared. In the Full Active case, all nodes of the network are active at all times. There is no sleep time. In the S-MAC case, all nodes sleep and wake up to send data at the same time in a synchronous manner independent of its position. In the DMAC/MTS case, each node wakes up in a predefined schedule dependent upon the node depth of the tree. In the DMAC/MTS, a node activates again five slots later if the node has more data to send or if a collision occurs.

In the proposed scheme, a TTS (Time Tree Scheduling) is used as in Equation (3) and (4). TTS is a scheduling scheme used in proposed Time Tree MAC. There are 10 independent branches in a network and two cases are considered. In TTS-2, ten branches are divided into two groups. One group wakes up at one time and the other group activates at next time. They are activated alternatively. In TTS-5, all branches are grouped in 5 groups, and activated per group in sequence. The sleep time of TTS-2 is same with DMAC/MTS in this simulation. The sleep time of the TTS-5 is 2.5 times longer than that of TTS-2.

### 4.2. Simulation Results

**Figure 7.**
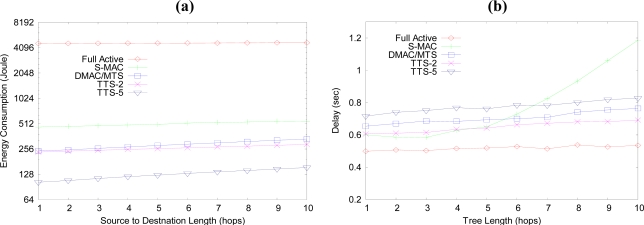
(a) Energy consumption of the network. (b) End-to-end delay characteristic with tree length.

Figure 7a is a graph of the energy consumption with varying distances from sources to destination. In this simulation, 10 nodes locate at the same depth of the tree and generate data traffic. The source traffic is light condition of CBR (Constant Bit Rate) with 1 second of interval time. The energy consumption and source distances are increased from the BS node; however, in the Full Active case, the difference due to the source depth is not large. Overall, the Full Active case consumes much more energy than the other cases. S-MAC consumes more energy than DMAC and TTS. The energy consumed by DMAC/MTS and TTS-2 is half of the energy consumed in S-MAC, and the energy consumption in TTS-5 is below half of the energy consumed in DMAC/MTS.

Figure 7b illustrates the characteristics of the end-to-end delay of a sensor network as with the tree length. At each simulation, the nodes that have same distance from the root node generate the data traffic, and the destination is the BS node. Normally, the end-to-end delay is not varied much with the hop counts of the tree. The delay is mainly part of the waiting time from the data generation time to the activation time of the node. Only a small bit of the delay is increased with increased tree length. The end-to-end delays are the least at the Full Active case. In this case, the data is sent as soon as generated. The delay in TTS-2 is a little lower than that of the DMAC/MTS. As the length of the hop counts between source and destination increases, the delay in S-MAC case increases fast after five hops while all other cases are not.

**Figure 8.**
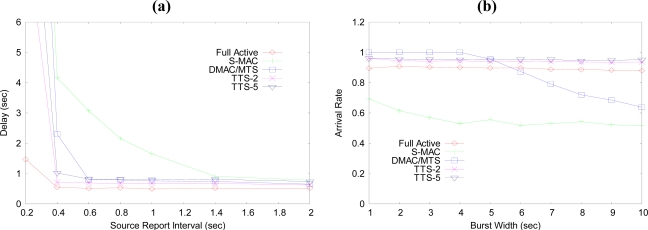
(a) Delay characteristics with source report interval. (b) Data arrival rate with burst width.

Figure 8a shows the delay characteristics of the sensor network seen by varying the data generation interval of the CBR source. When the source generation interval is over the value of 1.4, the delay of all the cases are near 1 second. The delay characteristic under 0.6 second is different at each case. In the Full Active case, the delay is the lowest value of all. The S-MAC case shows the longest delay characteristics. The TTS-2 and TTS-5 shows a lower delay than that of the DMAC/MTS case under the interval of 0.4 second of source traffic.

The data arrival rate of data to the BS node with varying the burst width is well shown in Figure 8b. In this simulation, the traffic load corresponds to the source interval of 1.0 second in Figure 8a. Under the burst width of 1.8 second, the arrival rate of each schemes except S-MAC case are similar. The Full Active, TTS-2 and TTS-5 cases are stable and not variant to the burst width. If the burst width goes up over the 5 seconds, the arrival rate of DMAC/MTS case goes down. The S-MAC case shows the worst performance in this simulation.

## 5. Conclusions

The proposed scheme uses a time scheduling scheme in data transfer at sensor networks based on a time tree. Each tree has different time schedules, so this scheme acts as a topology control. The sending time of a node is scheduled by the depth of the tree and has different times with the tree it included. The purpose of using a time tree is reducing the collision rate and the energy consumed at the sensor node. Compared to other scheduling schemes, the proposed scheme consumes less energy than DMAC/MTS and other schemes such as S-MAC and Full Active case. In addition, the delay and the arrival rate are stable compared to other schemes.

For applications that needs to report in a periodical manner, the proposed scheme can distribute the traffic to branches in a time tree destined by the target. This distributes energy consumed among nodes increasing the lifetime of the sensor network. To accomodate failures in sensor nodes, this scheme also delivers a minimum traffic to other branches of the time tree. Proposed retry and rate control schemes effectively reduce data transfer delay and increase data arrival rate.

The proposed medium access control scheme uses an RTS/CTS data transfer mechanism in the sensor network. The use of an RTS/CTS mechanism consumes more time and energy, but it reduces collision rates; furthermore, it provides the time adaptation mechanism between sensor nodes. In environments where many sensor nodes are densely deployed, our scheme sends data to the BS node more efficiently. The characteristics of the delay and arrival rate are also stable to the data traffic, especially to the burst traffics of the event monitoring in sensor networks.

## Figures and Tables

**Table 1.** Simulation parameters.

**Parameter**	**Values**	**Parameter**	**Values**

Network Areas	1,000 m × 550 m	Radio Bandwidth	1 Mbps
Radio Transmission Range	250 m	Transmission Power	0.66 W
Radio Inerference Range	550 m	Receive Power	0.395 W
Packet Length	100 bytes	Idle Power	0.35 W
Active Time	19.74 ms	Sleep Time	174 ms
